# Unlocking the distinctive enzymatic functions of the early plant biomass deconstructive genes in a brown rot fungus by cell-free protein expression

**DOI:** 10.1128/aem.00122-24

**Published:** 2024-04-03

**Authors:** Jesus D. Castaño, Irina V. El Khoury, Joshua Goering, James E. Evans, Jiwei Zhang

**Affiliations:** 1 Bioproducts and Biosystems Engineering, University of Minnesota, Saint Paul, Minnesota, USA; 2 Environmental Molecular Sciences Laboratory, Pacific Northwest National Laboratory, Richland, Washington, USA; 3 School of Biological Sciences, Washington State University, Pullman, Washington, USA; Shanghai Jiao Tong University, Shanghai, China

**Keywords:** fungal wood degradation, brown rot, ROS redox, fungal enzymes, wheat germ cell-free protein expression, biocatalyst consortium

## Abstract

Saprotrophic fungi that cause brown rot of woody biomass evolved a distinctive mechanism that relies on reactive oxygen species (ROS) to kick-start lignocellulosic polymers’ deconstruction. These ROS agents are generated at incipient decay stages through a series of redox relays that shuttle electrons from fungus’s central metabolism to extracellular Fenton chemistry. A list of genes has been suggested encoding the enzyme catalysts of the redox processes involved in ROS’s function. However, navigating the functions of the encoded enzymes has been challenging due to the lack of a rapid method for protein synthesis. Here, we employed cell-free expression system to synthesize four redox or degradative enzymes, which were identified, by transcriptomic data, as conserved players of the ROS oxidation phase across brown rot fungal species. All four enzymes were successfully expressed and showed activities that enable confident assignment of function, namely, benzoquinone reductase (BQR), ferric reductase, α-L-arabinofuranosidase (ABF), and heme-thiolate peroxidase (HTP). Detailed analysis of their catalytic features within the context of brown rot environments allowed us to interpret their roles during ROS-driven wood decomposition. Specifically, we validated the functions of BQR as the driver redox enzyme of Fenton cycles and reconstructed its interactions with the co-occurring HTP or laccase and ABF. Taken together, this research demonstrated that the cell-free expression platform is adequate for synthesizing functional fungal enzymes and provided an alternative route for the rapid characterization of fungal proteins, escalating our understanding of the distinctive biocatalyst system for plant biomass conversion.

**IMPORTANCE:**

Brown rot fungi are efficient wood decomposers in nature, and their unique degradative systems harbor untapped catalysts pursued by the biorefinery and bioremediation industries. While the use of “omics” platforms has recently uncovered the key “oxidative-hydrolytic” mechanisms that allow these fungi to attack lignocellulose, individual protein characterization is lagging behind due to the lack of a robust method for rapid synthesis of crucial fungal enzymes. This work delves into the studies of biochemical functions of brown rot enzymes using a rapid, cell-free expression platform, which allowed the successful depictions of enzymes’ catalytic features, their interactions with Fenton chemistry, and their roles played during the incipient stage of brown rot when fungus sets off the reactive oxygen species for oxidative degradation. We expect this research could illuminate cell-free protein expression system’s use to fulfill the increasing need for functional studies of fungal enzymes, advancing the discoveries of novel biomass-converting catalysts.

## INTRODUCTION

Brown rot fungi are saprotrophic decomposers capable of rapidly metabolizing carbon from the cell wall of dead woody tissues (1, 2). They selectively and efficiently degrade carbohydrates, while having a low preference for lignin mineralization (3–6). Their distinctive degradation mechanisms thereby hold valuable catalyst tools that can be innovated for the efficient manufacturing of “chemical blocks” and bio-based products (7–10). It has been previously uncovered that fungi achieve this by consolidating the non-enzymatic lignocellulose depolymerization that relies on reactive oxygen species (ROS) for pretreatment attacks (e.g., electrophilic addition or hydrogen abstraction) and the enzymatic degradation that is mediated by glycoside hydrolases to depolymerize poly/oligo-saccharides (6, 11–17). The •OH radical generated via the Fenton reaction (Fe^2+^ + H_2_O_2_ → Fe^3+^ + •OH + OH^−^) is at a central place for driving non-enzymatic degradation, and its aggressive attack is generally believed to be the primary cause of the fast brown rot decay (11, 12). Despite the importance of the non-enzymatic oxidative processes in brown rot, their biological pathways are still understudied, and their interaction with the hydrolytic enzymes remains unclear.

Two routes are likely involved in brown rot for generating Fenton reactants—Fe^2+^ and H_2_O_2_, by either oxidoreductases or low-molecular-weight (LMW), redox-active metabolites-mediated reductions of Fe^3+^ and O_2_ (18, 19). The first route involves a collective of auxiliary activities (AA) oxidoreductases capable of enzymatically reducing O_2_ and Fe^3+^. These include the H_2_O_2_-producing glucose-methanol-choline (GMC) oxidoreductases in AA3, AA4, and AA5 families (20, 21) and the ferric reductase in cytochrome domain-containing AA8 family (22) that were documented in the CAZy database (Carbohydrate-Active enZYmes; http://www.cazy.org/). The second route involves shuttling electrons from the central cellular metabolic pathway to secreted LMW reductants, and then to O_2_ and Fe^3+^ in the vicinity of lignocellulose chains, thus initiating the localized Fenton attack (12, 23). A diverse group of LMW reductants have been isolated and characterized from different brown rot fungal lineages, with dimethoxyhydroquinone (DMHQ) as the most well-studied one (24–28). By the diffusion of DMHQ to the extracellular matrix, brown rot fungi can shuttle the intracellular electrons (e.g., NAD(P)H) to the wood substrate for oxidative degradation, and the oxidized format DMBQ is then recycled by the putative benzoquinone reductase (BQR; AA6 family), thus sustaining a redox cycle (Reactions 1–8) (19, 29, 30). An array of genes (i.e., Fenton genes) encoding putative enzyme functions for both routes have been discovered across different brown rot fungi, yet their physiological roles in mediating Fenton reaction and the non-enzymatic attacks remain undetermined.


(1) $$DMHQ+O_{2}\to DMHQ\;∙+∙\;O_{2}^{-}+H^{+}$$

(2) $$DMHQ\;∙+O_{2}\to DMBQ+∙\;O_{2}^{-}+H^{+}$$

(3) $$DMHQ+{Fe}^{3+}\to DMHQ\;∙+{Fe}^{2+}+H^{+}$$

(4) $$DMHQ\;∙+{Fe}^{3+}\to DMBQ+{Fe}^{2+}+H^{+}$$

(5) $${Fe}^{3+}+∙\;O_{2}^{-}⇌{Fe}^{2+}+O_{2}$$

(6) $$2\;∙\;O_{2}^{-}+2H^{+}\to H_{2}O_{2}+O_{2}$$

(7) $$Fe^{2+}+H_{2}O_{2}⟶Fe^{3+}+HO^{-}+∙\;OH$$

(8) $$DMBQ+NAD(P)H\overset{BQR?}{⟶}DMHQ+NAD(P{)}^{+}$$

$$\left(X\;∙\;represent\;intermediate\;radicals\;shuttling\;e^{-}\;to\;∙\;OH\right)$$

Characterizing the functions of these plausible Fenton genes remains challenging due to the absence of well-established, rapid protein expression platforms for fungal enzymes, as well as the presence of a vast number of candidate genes to test. For instance, till October 2023, 17 families of auxiliary redox enzymes with more than 25,000 genes have been documented in the CAZy database, and among these at least six families (AA3–8) and some LPMOs are associated with the generation of Fenton reactants (http://www.cazy.org/Auxiliary-Activities) (22). Regarding each brown rot species, there are about 20–40 genes on average in these six Fenton AA families, with some families comprising multiple homologous genes (https://mycocosm.jgi.doe.gov/mycocosm/home). Although new tools have significantly advanced sequence-based protein function prediction (31–33), it is still difficult to discern between specific enzymatic functions among homologous proteins that belong to the same protein family and share a highly similar structural domain. Thus, informatics-dependent approaches may have limitations in determining a gene’s function, unless its encoded enzyme is biochemically characterized. Also, the protein function prediction tools do not provide information on interactions of enzymes in a protein consortium and with surrounding environments, making it challenging to interpret the enzyme’s “*in situ*” functions in the specific context of brown rot environments. There are evidences that some Fenton genes, side-chain acting hemicellulases, and ligninolytic enzymes were co-expressed at the oxidation step of brown rot (6, 34, 35), suggesting that these enzymes likely interact with each other or with the surrounding harsh radical environment. Overall, these challenges are begging new protein expression tools that can allow characterization, at a large scale, of the functions of brown rot degradative genes/enzymes, as well as their possible interactions in the context of the unique fungal oxidative environments.

The cell-free expression system allows rapid protein synthesis compared to the conventional recombinant protein expression strategies, and it is therefore becoming a workhorse for many structural and functional validation studies of enzymes from different origins (36). Specifically, this strategy offers advantages such as no need for codon optimization, increased protein folding, solubility, and overall yields, and shortened processing time, which in combination are expected to empower high-throughput protein synthesis for characterization purposes (36–38). Also, cell-free protein expression systems are especially advantageous for the expression of toxic proteins, and they offer flexibility in co-factor supplementation according to users’ needs (36–38). Hence, its co-expression conditions for the synthesis of hetero-complexes can be rapidly adjusted by simple combinatorial mixing of single gene-harboring plasmids to study increasingly complex macromolecular systems (39). Considering that fungal protein families remain largely unexplored, it will be valuable to investigate cell-free protein expression as an alternative approach for the characterization of fungal protein functions, thus advancing the large-scale mining of untapped fungal biocatalysts.

Despite the fact that comparative genomic and transcriptomic analyses have, to some extent, provided clues to help link Fenton genes to their brown rot functions (6, 16, 34), a universal Fenton gene has not been functionalized. In this regard, it would be useful to interrogate the protein functions, gene-by-gene, using a rapid protein expression method. In this work, we employed a cell-free expression system to rapidly synthesize candidate degradative enzymes whose transcriptional roles were putatively circumscribed to the oxidative stage of wood decay and were conserved across brown rot fungal species. The functional characterization of these synthesized enzymes then allowed the interpretation of their roles in mediating the oxidation process of brown rot. Importantly, it allowed the discovery of the interactions of Fenton chemistry with its co-existing lignocellulolytic enzymes. Thus, these results suggested that the cell-free expression system can be an alternative method to quickly synthesize and characterize fungal enzymes for discovering novel biomass-converting catalysts.

## RESULTS AND DISCUSSION

### Candidate fungal enzymes involved in the oxidative step of brown rot wood decomposition

**Fig 1:**
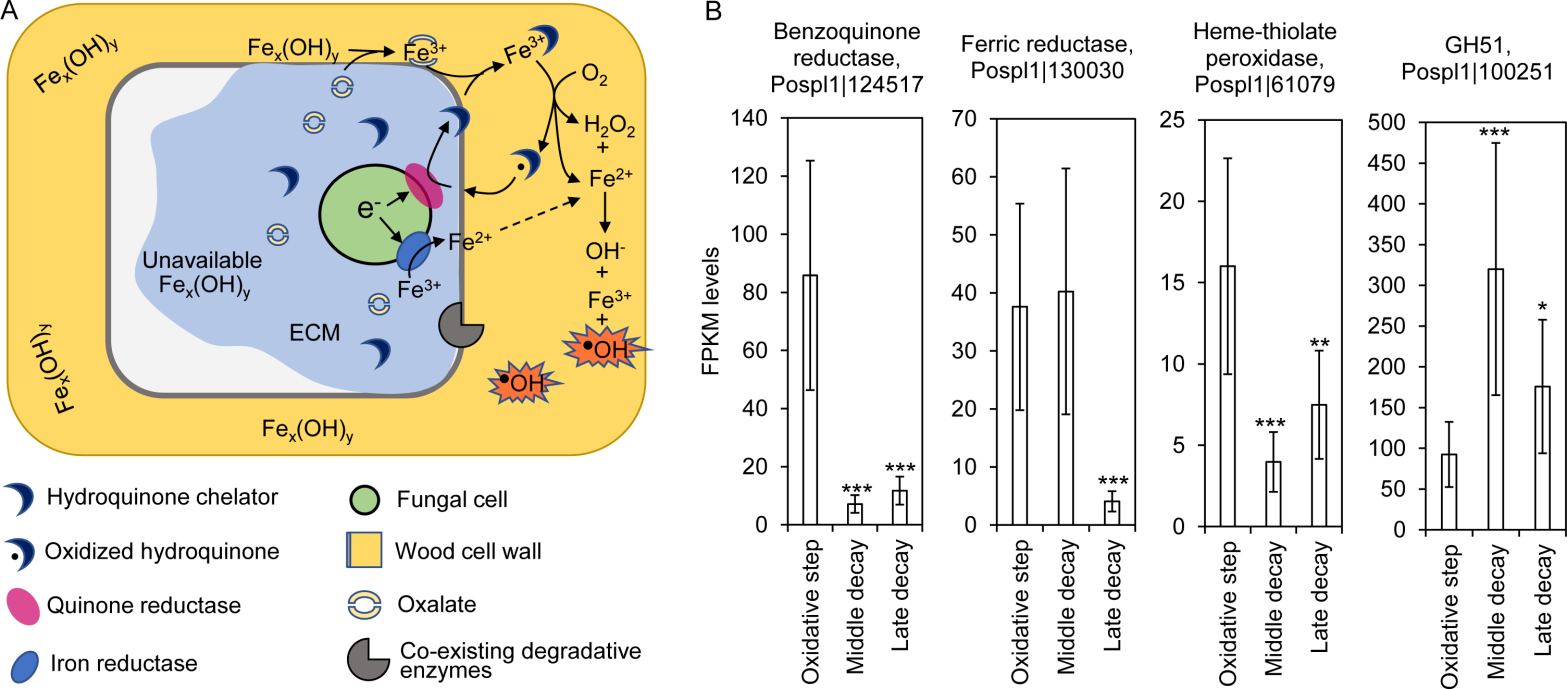
Candidate CAZy enzymes involved in the brown rot fungal oxidative deconstruction of wood cell wall. The fungal enzyme consortium, chelator metabolites, and their mediated redox reactions for generating Fenton reactants, Fe^2+^ and H_2_O_2_, were proposed for the oxidative step of brown rot according to the previous research (**A**) (6, 16). The putative enzyme catalysts in this model included Fenton-associating oxidoreductases (e.g., benzoquinone reductase and ferric reductase) and their synchronistic enzymes (e.g., heme-thiolate peroxidase and hemicellulose-debranching glycoside hydrolase in the GH51 family). The upregulation or high expression of four corresponding genes at the early oxidation stage of *Rhodonia placenta*, relative to the advanced decay stages (middle and late decay), suggested that they play a role in the oxidative decomposition of wood cell wall (**B**). Gene expression data were retrieved from the data set of GSE84529 (6), and the expression of the four candidate genes was presented as FPKM mean values ± SD of three bio-replicates. Gene codes were taken from the JGI database *R. placenta* MAD 698R v1.0 (https://mycocosm.jgi.doe.gov/Pospl1/Pospl1.home.html). ECM (light blue) represents the fungal extracellular matrix mediating the diffusion of enzymes and metabolites. Significant differences of gene expressions between the oxidation and advanced decay stages were indicated by the FDR values of RNA-seq DEG analysis (*, FDR < 0.05; **, FDR < 0.01; ***, FDR < 0.001).

Brown rot fungi use a ROS-dependent, metal- and LMW metabolite-mediated oxidative mechanism to kick off wood degradation (16, 34, 40). Subsequently, the released oligosaccharides are hydrolyzed with glycoside hydrolases (6, 16). Although the oxidative step plays a central role in the entire brown rot process, the biological pathways responsible for it are still not well understood. Aided by a temporal transcriptomic analysis across model brown rot species *Rhodonia placenta* and *Gloeophyllum trabeum* (6), we identified conserved ROS- and ROS-partnership genes involved in the oxidative step by identifying if their transcription levels were pronouncedly upregulated or at high levels (for ABF) at the early stages of brown rot (Fig. 1A). Among these genes, we found that encode benzoquinone and ferric ion reductases (BQR and FRD, respectively), which are important to keep the Fenton reaction cycle active by helping reduce Fe^3+^ (5). Besides Fenton-associated genes, we also isolated other co-expressed genes encoding heme-thiolate peroxidase (HTP) involved in unspecific hydroxylation of aromatic rings (41), and early glycoside hydrolases such as an α-L-arabinofuranosidase (ABF) in family GH51 that possessed high expression levels and may act in partnership with the Fenton mechanism to hydrolyze lignocellulose (6). In *R. placenta*, the protein IDs of these four CAZymes were identified as Pospl1|124517/Pospl1|64069 (two alleles) for BQR, Pospl1|130030 for FRD, Pospl1|61079/Pospl1|25391 for HTP, and Pospl1|100251/Pospl1|127046 for ABF, respectively. As the two alleles showed the same expression patterns, here only one allele was presented for each enzyme, as in Fig. 1B. Given the highlighted roles of these candidate genes in the oxidative step of brown rot, in this research, we characterized their enzyme functions by cell-free system-aided protein expression.

### Brown rot fungal enzymes were synthesized by the cell-free expression

**Fig 2:**
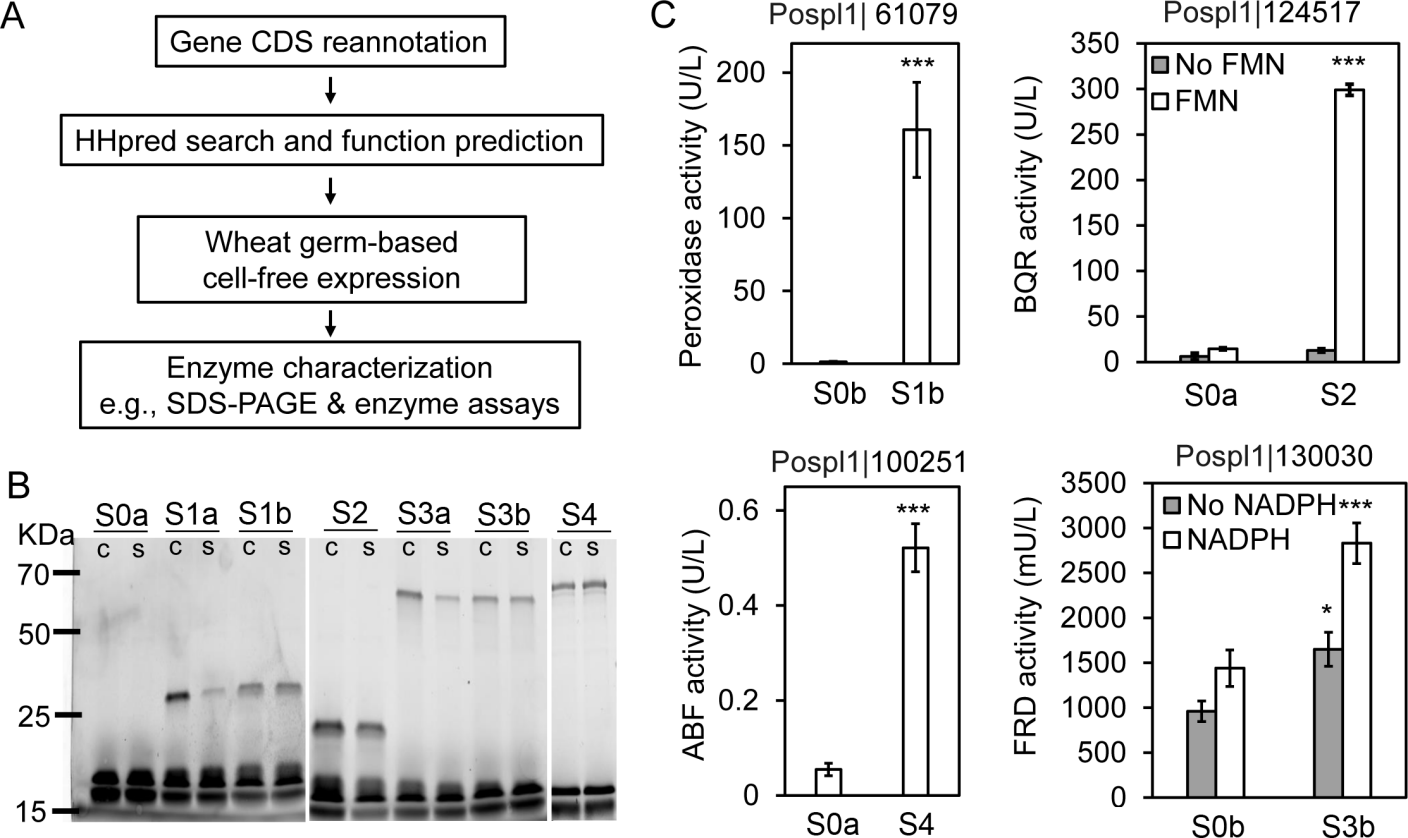
Cell-free synthesis and preliminary functional characterization of brown rot fungal enzymes. A pipeline from CDS annotation to cell-free protein expression and characterization was used for the study of brown rot fungal enzymes (**A**). DNA constructs containing the CDS of interest were transcribed and translated using wheat germ-based cell-free expression in a screening mode with the use of a FluoroTect Green_Lys_ reagent—a method described previously (36). Successful expression and solubility of target proteins were then visualized on SDS-PAGE using a laser-based scanner (**B**). Symbols “c” and “s” refer to the crude and soluble fractions of each cell-free protein sample, respectively. The FluoroTect Green_Lys_ reagent itself manifests as two dark bottom bands in every lane of the gel. The subsequent enzyme assays showed that the synthesized fungal proteins Pospl1|61079, Pospl1|124517, Pospl1|130030, and Pospl1|100251 had the expected heme-thiolate peroxidase, benzoquinone reductase, α-L-arabinofuranosidase, and ferric reductase activities, respectively (**C**). Mean values ± SD of three bio-replicates were shown for the enzyme assays, and two-tailed paired *t* tests were used to establish significant differences between the sample and its corresponding control (*, *P* < 0.05; **, *P* < 0.01; ***, *P* < 0.001). The list of protein sample names and their relevant information can be seen in Table 1.

Prior to expressing proteins from the corresponding candidate fungal genes, their CDS regions were re-annotated by integrating transcriptomic evidence, and this resulted in an improved definition of “exon-intron junctions” in the gene models of ferric reductase and α-L-arabinofuranosidase (Fig. S1). The new CDS sequences were then sent into the pipeline for protein function prediction and expression (Fig. 2A; Table 1). The *in-silico* analysis showed that all of the four candidates were predicted as having a unitary enzyme function.

**TABLE 1:** List of proteins that were synthesized using the cell-free expression in this study

JGI ID^*a*^	New ID	Sample name^*b*^	Size (kDa)^*e*^	Predicted protein function^*f*^
N/A	N/A	S0a, S0b^*c*^	0	Control without protein of interest
Pospl1|61079	g9701	S1a^*d*^, S1b^*c*^	32	Heme-thiolate peroxidase; EX w/ SignalP
Pospl1|124517	g9613	S2	25	Benzoquinone reductase; IN
Pospl1|130030	g11221	S3a, S3b^*d*^	69	Ferric reductase; TM
Pospl1|100251	g3363	S4	68	GH51 α-L-arabinofuranosidase; EX w/ SignalP

^*a*^ IDs of target proteins were obtained from JGI *Rhodonia placenta* MAD 698-R v1.0 (https://mycocosm.jgi.doe.gov/Pospl1/Pospl1.home.html), and new IDs are from the re-annotated genome of our previous research and are available in the database GSE108189 (6).

^*b*^ Synthesized protein samples under different cell-free protein synthesis conditions. Negative controls S0a and S0b correspond to the wheat germ-based expression reactions conducted in the absence of DNA template (pEU vector).

^*c*^ Protein samples synthesized by supplementing nanodiscs and hemin.

^*d*^ Protein samples synthesized in the presence of nanodiscs.

^*e*^ Protein size was predicted based the amino acid sequence.

^*f*^ Protein functions were predicted by InterProScan and HHpred. DeepTMHMM was used for the prediction of transmembrane topology and signal peptide (SignalP), with EX, IN, TM represent extracellular, intracellular, and transmembrane proteins, respectively.

To enable the rapid protein synthesis for enzyme characterization, a eukaryotic, plant-based (wheat germ) cell-free expression system was applied in this work. Using this method, we successfully expressed all four candidate proteins, demonstrated by a fluorophore-labeled SDS-PAGE (Fig. 2B). All of the expressed proteins exhibited the predicted enzyme activities relative to the control, namely, heme-thiolate peroxidase (HTP) (Sample S1b; Protein ID Pospl1|61079), benzoquinone reductase (BQR) (S2; Pospl1|124517), ferric reductase (FRD) (S3b; Pospl1|130030), and α-L-arabinofuranosidase (ABF) (S4; Pospl1|100251) (Fig. 2C). Among these, the activities of BQR and FRD were highly dependent on the presence FMN and NADPH as cofactors, respectively (42, 43). In addition, we found that adding nanodiscs was particularly instrumental in this experiment for the synthesis and correct folding of an active FRD, which is a prosthetic hemin group-containing protein predicted to have a short transmembrane helix (44). Notably, despite the fact that plant-based cell-free expression systems have a limited ability to introduce post-translational modifications (PTMs; e.g., glycosylation) that are often required for proper folding/function of select fungal enzymes (e.g., glycoside hydrolase) (45, 46), this did not influence the catalytic functions of the four degradative enzymes tested in this work. However, to know whether glycosylation will influence the stability of the cell-free synthesized proteins, more research is required. Taken together, the success on obtaining four active brown rot enzymes allowed us to interrogate their biochemical roles in mediating the fungal decomposition of wood.

### Enzyme catalytic features under varying environmental conditions may reflect their roles in brown rot wood decay

**Fig 3:**
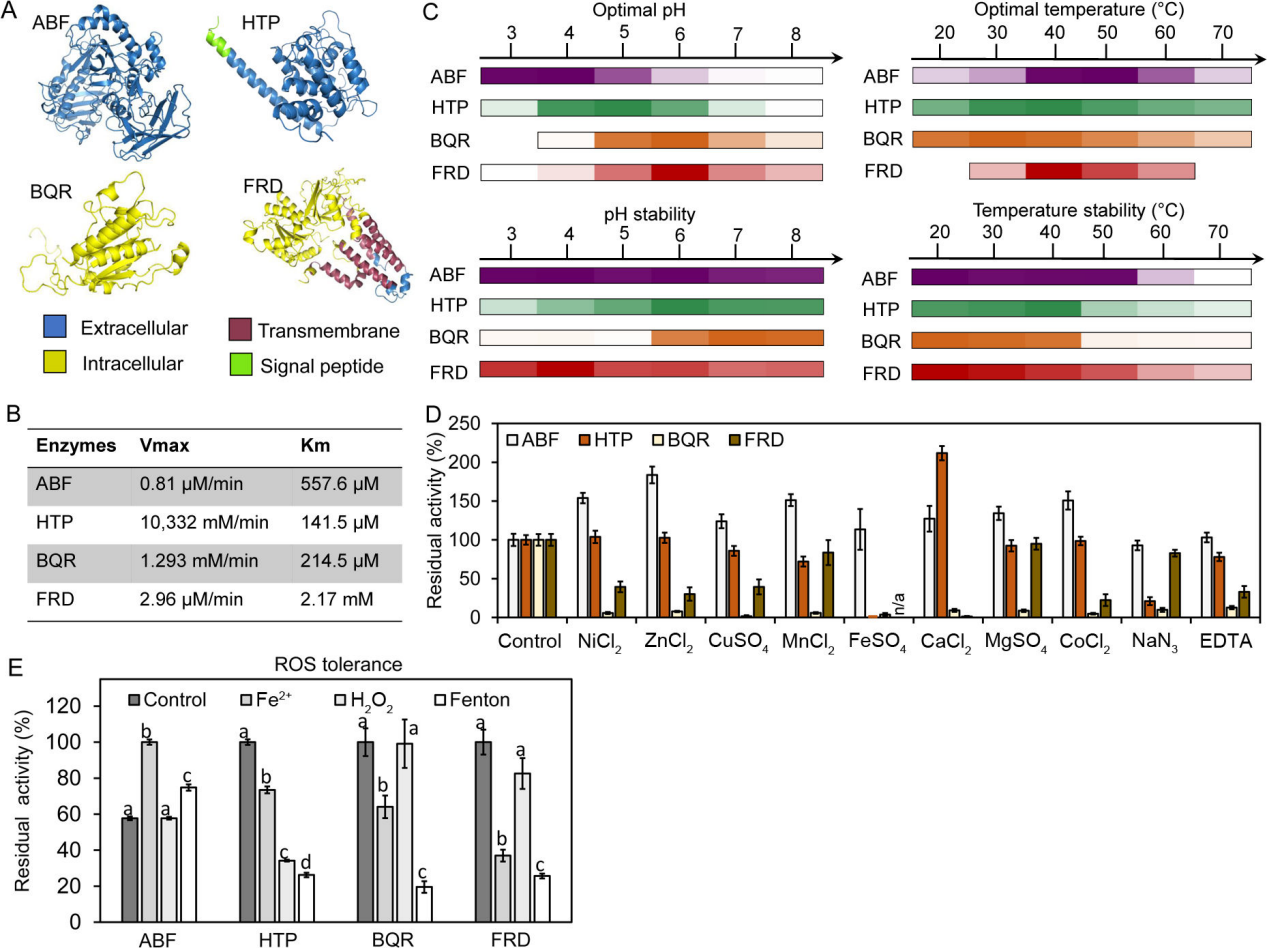
Protein structures, catalytic features, and responses to environmental factors of the synthesized brown rot fungal enzymes. The 3D protein structures and their possible cellular loci were predicted for four active brown rot enzymes—ABF, HTP, BQR, and FRD (**A**). Kinetic parameters, including the maximum velocity (*V*_max_) and *K*_*m*_ values, were evaluated for each enzyme (**B**). Catalytic features, including the optimal pH, pH stability, optimal temperature, and temperature stability of four enzymes, were determined and presented in the format of heatmap, with a higher color intensity indicating a higher activity in each case (**C**). The numerical data set of these measurements is available in Fig. S2 to S5. Potential environmental inhibitors (**D**) and the effect of oxidative stress (**E**) on enzyme activities were also evaluated. The effects of oxidative stress were measured by monitoring the enzyme tolerance to the ROS radicals (•OH) generated by the Fenton reaction (Fenton) relative to that of control (i.e., without both H_2_O_2_ and Fe^2+^) and Fe^2+^- or H_2_O_2_-only treatments (1 mM Fe^2+^ and 5 mM H_2_O_2_ were used for these experiments, and see more details in the methods for the Fenton treatment). All enzyme activities were shown as the mean values ± SD of three replicates, and different letters represent significant differences within a series of treatment for each enzyme (*P* < 0.05).

The early stage of brown rot is often accompanied by the fungal-amended increases in the levels of H^+^ and metal availability that are boosted by the secretion of organic acids (e.g., oxalic acid), the elevated oxidative potentials caused by the production of ROS, and other environmental traits that can allow oxidative attacks on wood substrates (40, 47–50). These environmental changes not only act as signals to trigger fungus’ physiological responses (35, 51), but they could also interact with fungal enzymes via controlling the reaction conditions or modifying the functional groups in proteins (52–55). Therefore, measuring the effects of environmental variables on catalytic functions would guide the understanding of enzyme roles in brown rot lignocellulose degradation. In this regard, after confirming the activities of the cell-free expressed enzymes, we proceeded to characterize their catalytic properties in response to different environmental factors (e.g., pH, temperature, metals, and ROS) that are crucial to the early stages of brown rot (Fig. 3).

**Fig 4:**
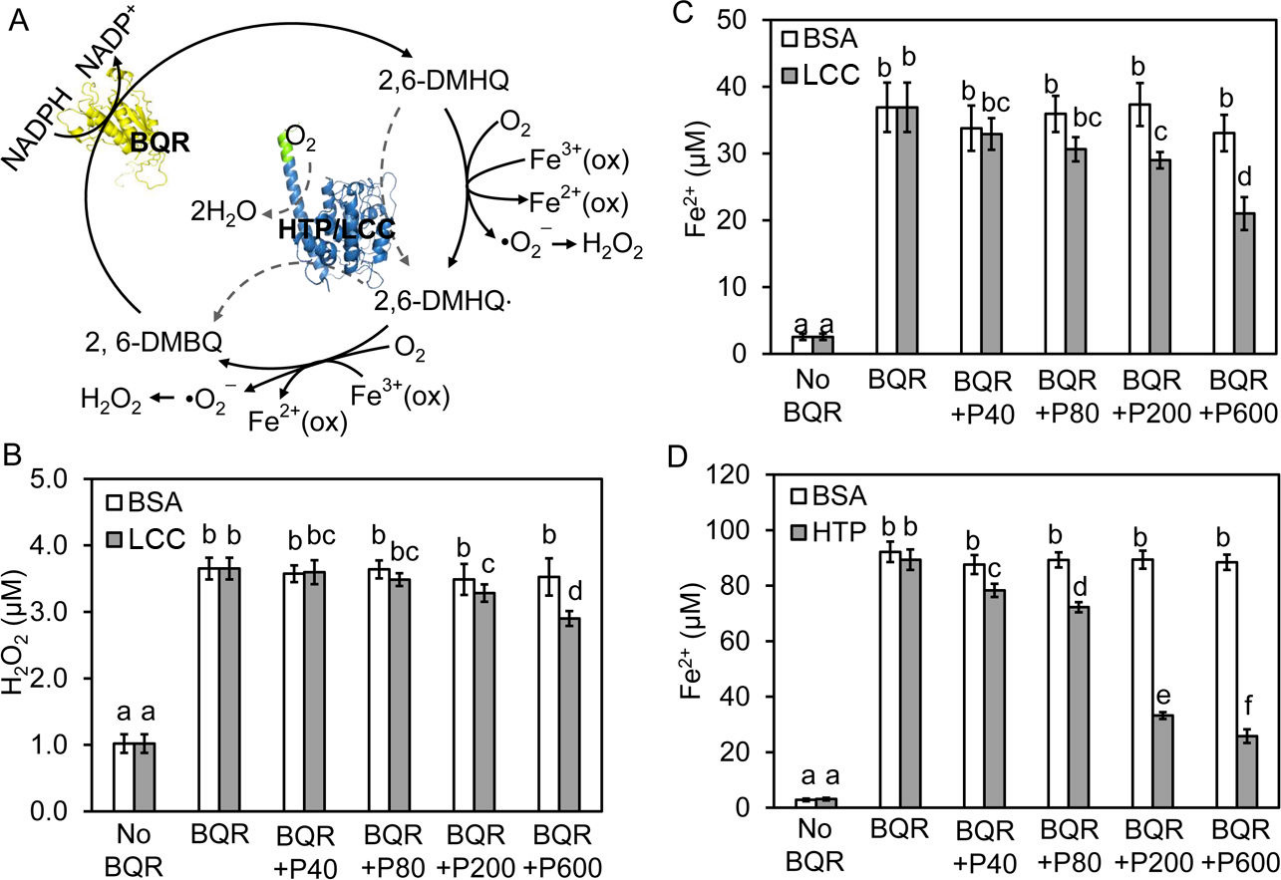
The influences of phenol-oxidizing enzymes on quinone redox cycle that drives Fenton chemistry in brown rot. (**A**) The proposed quinone redox cycle driven by a BQR of *R. placenta* and its partner oxidases: laccase (LCC; Sigma, SIAL-SAE0050) and heme-thiolate peroxidase (HTP). In line with the proposed reactions, the reduction of 2,6-DMBQ by BQR allowed the detection of Fe^2+^. Additionally, adding LCC to the cycle caused a protein dosage-dependent interference with the generation of both H_2_O_2_ (**B**) and Fe^2+^ (**C**). Similarly, adding HTP also lowered the production of Fe^2+^ (**D**). A higher amount of Fe^3+^ was used for the HTP supplemental experiments to increase the detection limits of Fe^2+^, given that adding exogenous H_2_O_2_, required by HTP activity, may accelerate the oxidative consumption of generated Fe^2+^. Different amounts of LCC or HTP proteins were supplemented into the reactions, including 40 µg (+P40), 80 µg (+P80), 200 µg (+P200), and 600 µg (+P600). Bovine albumin serum (BSA) was used as a control in both cases to account for any unspecific scavenging effects. Mean values ± SD of three bio-replicates were shown for the production of H_2_O_2_ and Fe^2+^. Different letters represent significant differences within a series of treatment (ANOVA, *P* < 0.05).

As a proposed quinone redox driver enzyme for Fenton chemistry (56)—the BQR—in our assays showed an optimum catalytic pH of 6.0 and a strong stability around neutral pH, while it was pronouncedly sensitive to environmental stresses including pH levels below 5.0, temperatures above 40°C, several metal inhibitors, and a harsher oxidative environment generated by •OH but not H_2_O_2_ (Fig. 3C through E; Fig. S2). These features of BQR suggested it was most likely an intracellularly located enzyme and thereby sheltered from the unfavorable brown rot environments such as the low acidic pH (<4.2) and high metal solubility (57). Our protein modeling results (Fig. 3A), as well as the previous studies (24, 56), supported that BQR is an intracellular enzyme. The following measuring in Fig. 4 further confirmed that BQR is an enzyme capable of generating reduced quinones and is sufficient to drive the production of both Fenton reactants (*as in the following section*). Nonetheless, its cytoplasmic localization implies the presence of an efficient transport or diffusion mechanism that enables the redistribution of reduced quinones for extracellular Fenton chemistry, an area that remains unexplored.

The FRD studied in this work is a membrane-bound enzyme as indicated by its seven transmembrane domains (Fig. 3A) (6). Similar to BQR, this enzyme had an optimum catalytic pH of 6.0 and was sensitive to some metal and EDTA inhibitors. However, its activity was resilient to a broad range of pH and temperature conditions likely due to the stable membrane-bound structures (Fig. 3C through E; Fig. S3) (58). Based on its membrane-bound features, we deduced that FRD might play two roles during the early brown rot stage: (i) Maintaining the iron homeostasis through generating reduced iron for fungal cell uptake. In this regard, two iron permeases were discovered that were co-upregulated together with this FRD at the early brown rot stages (16); (ii) Providing reduced iron for extracellular Fenton chemistry. The reduced iron, instead of being immediately oxidized, could be chelated by the high concentration of oxalate in the vicinity of fungal cells. Thus, a balance between intracellular uptake and extracellular diffusion will likely determine the trajectory of reduced iron and thence the roles of FRD in brown rot. Nonetheless, more molecular and genetic evidence is required to further validate FRD roles in brown rot.

In contrast to BQR and FRD enzymes, our assays revealed that both ABF and HTP exhibited the features of typical fungal lignocellulolytic hydrolases, with optimal catalytic pH levels between 4 and 5, along with an optimal temperature range between 40°C and 50°C. This aligns with the typical characteristics of biomass-degrading enzymes (Fig. 3C; Fig. S4 and S5) (59–61). Protein modeling showed that both enzymes were located in the extracellular environment where the production of oxalate often drives the pH below 4.2 (47), which is in line with their requirements of an acidic catalytic environment (Fig. 3A). Despite sharing some catalytic features, the two enzymes showed different pH stabilities, with ABF being remarkably stable at a broad range of pH levels, while HTP was readily susceptible to pH levels lower than 3.0 (Fig. 3C). Also, HTP was sensitive to temperatures >40°C, Fe^2+^ and NaN_3_ inhibitors, and H_2_O_2_. This is probably due to damage caused by reactive azidyl/oxygen radicals that can oxidize tryptophan residues in the enzyme (52, 53, 55, 62). Additionally, HTP activity was boosted by the presence of Ca^2+^. While calcium has been identified as essential for peroxidase activity before (63), with some peroxidases even containing calmodulin domains (64, 65), its role is not completely understood yet. Similarly, for ABF, all the ions tested—except for Fe^2+^—significantly increased the enzyme activity, but neither EDTA nor NaN_3_ showed any effects. The great robustness of ABF to various environmental stresses indicated that it has evolved special features to adapt to the unique brown rot mechanisms.

### Resilience of the fungal α-L-arabinofuranosidase (ABF) to oxidative stress

The cell-free synthesized enzyme allowed us to discover a hemicellulose-debranching glycoside hydrolase, ABF, that was tolerant to a variety of brown rot-associated environmental stresses, particularly to the Fenton chemistry. Evidently, this ABF exhibited a stronger resistance to the overall effects of Fenton reaction, while the other three synthesized enzymes had approximately 80% of the initial activity diminished after the Fenton treatment (Fig. 3E). Likely, the Fenton damages were delivered by either the •OH radicals (e.g., BQR and FDR) or the additive effects of H_2_O_2_ and Fe^2+^ (e.g., HTP), as the previous studies showed that the oxidative modifications on amino acid residues such as tyrosine or tryptophan can result in protein aggregation, catalytic domain disruption, and ultimately the loss of enzyme functions (66–68). With regard to ABF, the H_2_O_2_-only treatment showed no damaging effects, measured by monitoring the residual activities after oxidation treatment, while Fe^2+^ boosted the enzyme activity; thus, it is possible that the boosting effect of Fe^2+^ may have compensated the damages caused by •OH radicals. Although the activating mechanism of Fe^2+^ was not clear, the tolerance exhibited by ABF toward ROS and the Fenton reagents was exceptional, and this is consistent with our previous findings by using the crude enzymes prepared from *R. placenta* growing on wood substrates (47, 69). RNA-seq analysis indicated this ABF was expressed at high levels at the early stages of brown rot while the ROS attack sets in and causes initial damage to the lignin barrier to expose more hemicellulose fibers (70); thus, its interaction with Fenton chemistry seems inevitable. Here, the discovery of the ABF’s resilience to oxidative stress further suggests that fungi have co-evolved sturdy structures to cope with its adaptation of using ROS for fast wood decomposition.

### Enzyme interactions with the quinone redox cycles

It has been proposed that the quinone-hydroquinone cycle driven by BQR is a key mechanism contributing to the extracellular Fenton chemistry of brown rot (24, 26, 27, 56). However, there are still complex interactions that have not been elucidated. Some phenol-oxidizing enzymes, such as laccase and heme-thiolate peroxidases, are co-expressed with BQR during the early brown rot stages (6, 16, 71), and they may oxidize hydroquinones and thereby influence the electron transfer to Fe^3+^ or O_2_ (Fig. 4A). For example, among the five laccase-like genes in *R. placenta,* Pospl1|47589 showed an upregulation by 1.8-fold at the early brown rot stage (16), and similar early upregulation was also evidenced for the heme-thiolate peroxidase Pospl1|61079 (Fig. 1B). Although their coexistence with ROS is clear, the roles that these oxidases play in quinone redox cycling remains unclear. Here, using the cell-free synthesized BQR to drive the reduction of 2,6-dimethoxybenzoquinone (2,6-DMBQ) to 2,6-dimethoxybenzohydroquinone (2,6-DMHQ), we first built an *in vitro* quinone redox cycle that was able to effectively produce Fe^2+^, as well as H_2_O_2_ (Fig. 4B and C), while the non-BQR controls were not positive for both Fenton reactants. We then evaluated the effects of adding HTP or laccase to this system by monitoring how they could influence the production of Fenton reactants. Our results suggested that both enzymes inhibited the production of Fe^2+^ or H_2_O_2_ compared to the control BQR mixture with no laccase or HTP added (Fig. 4B through D). Also, the results suggested that this inhibitory effect was enzyme dosage-dependent, in which the inhibition rates were positively correlated with the amounts of the supplemented laccase/HTP enzymes.

**Fig 5:**
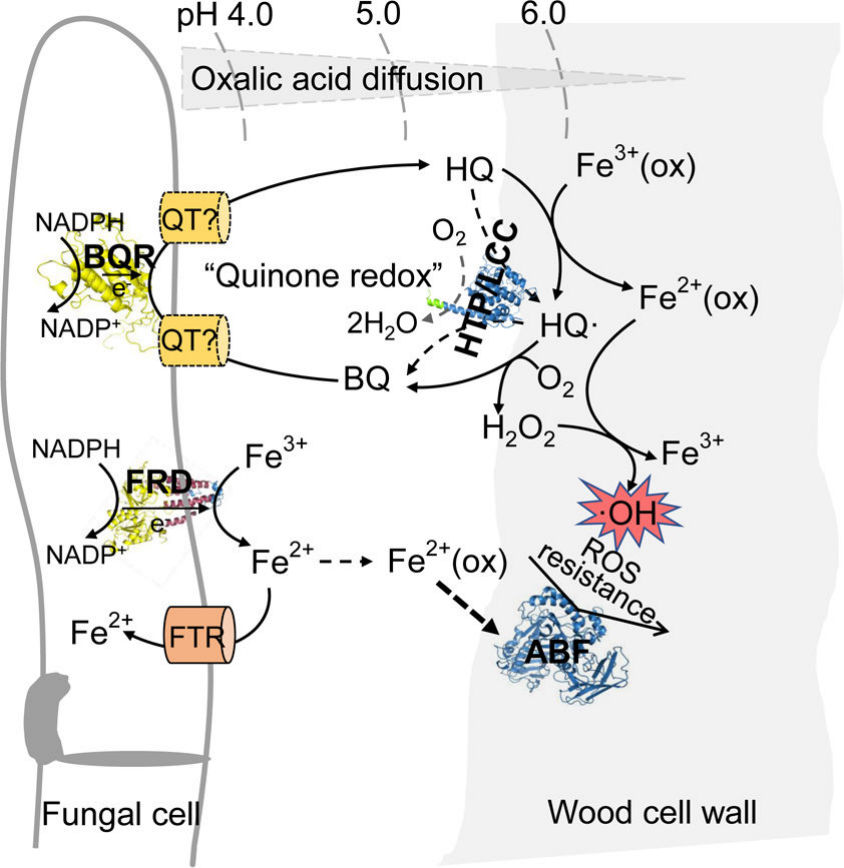
Schematic model depicting the functions of fungal enzymes during the oxidative phase of brown rot wood decomposition. Four characterized enzymes by this work, as well as their microenvironments (e.g., oxalate and pH) between the fungal cell and wood substrate, were included to build this model. Briefly, the benzoquinone reductase (BQR) located inside the fungal cell drives the quinone redox cycle, thus translocating intracellular reducing equivalents to drive the extracellular reduction of Fe^3+^ and O_2_ for Fenton reaction on wood substrate. A quinone transporter (QT), despite not yet being characterized, is expected in this process to facilitate the diffusions of the quinone metabolites. Microenvironments composed of decreasing concentrations of protons and oxalate, formed while they are diffused from fungal cell to wood, are required to control the localized Fenton reactions adjacent to the wood substrate. Marginal production of ligninolytic oxidases [e.g., laccase (LCC) and heme-thiolate peroxidase (HTP)] by brown rot fungi may slow down the generation of Fenton radicals produced by quinone redox molecules. Bi-functionility is proposed for ferric reductase (FRD) in the model, which includes maintaining iron homeostasis and production of reduced iron to facilitate wood decay processes. Synergy between GH catalysts and Fenton mechanism is evident, but they may have evolved distinct structural or catalytic features to resist the ROS damage. For instance, in this study a GH51 family α-L-arabinofuranosidase (ABF) displayed outstanding ROS-tolerance, and it may be involved in debranching hemicellulose immediately after ROS depolymerization, thus exposing the cellulose chains.

Notably, our findings here are different from that of Wei et al. (25), who found an enhancing effect, but not inhibition, of laccase on the quinone redox cycle. In their research, the authors assumed that during brown rot the Fe^3+^ was fully chelated by oxalate to form the Fe^3+^-trioxalate (Fe(ox)_3_^3−^) complex, making its oxidative potential too low to be directly reduced by hydroquinone, and thus a laccase-mediated catalytic step would therefore be required to first turn hydroquinones into the semiquinone radicals that can more readily reduce the chelated Fe^3+^, given their more negative reduction potentials (56). In our assays, relatively lower oxalate levels were applied after considering the diffusion effect of this fungal metabolite in the fungal extracellular matrix and wood substrate (Fig. 5) (72). In short, we used oxalate:Fe^3+^ molar ratio of 10:1 at pH 5.0, and under these conditions Fe^3+^ primarily occurred as Fe^3+^-monooxalate (Fe(ox)^+^) (E^°^ = 0.43 V) that can directly oxidize hydroquinones (58, 73). Thus, our quinone redox system manifested a capacity that was sufficient enough to reduce Fe^3+^ chelated by oxalate without the need of laccase catalysis. Furthermore, when laccase or HTP was overloaded, the 2,6-DMHQ would be rapidly oxidized to 2,6-DMBQ with the concomitant net production of H_2_O rather than •O_2_^¯^ (74), and this process would outcompete that of Fe^3+^ reduction by semiquinone 2,6-DMHQ· (*dash line indicated paths in* Fig. 4A). Taken together, we concluded that, by redirecting electrons to reduce O_2_ to H_2_O, phenol-oxidizing enzymes were likely “braking” the speed of Fenton reactants’ production in the quinone redox cycles.

Our findings of the interactions between ligninolytic oxidases and quinone redox cycles may provide new insights to interpreting the distinctive degradative systems adapted by different decay manners—white and brown rot. For a long period, it is defaulted that white rot fungi express a large quantity of ligninolytic oxidases (e.g., laccase and ligninolytic peroxidases) to break down and modify lignin barriers to get access to carbohydrates, while brown rot fungi use the non-enzymatic mechanism that relies on Fenton cycles to degrade lignin (1, 2, 11). Phylogenomic studies suggested that the brown rot mode was evolved from white rot ancestors by abandoning most of the ligninolytic genes and maintaining only a marginal laccase activity (2, 75, 76). Besides gene losses, functional genomic comparisons suggested that brown rot adapted most of the ancestral oxidoreductase genes from white rot as its non-enzymatic machinery (6, 20, 21). However, this did not explain why the non-enzymatic oxidation was distinctively adopted by brown rot, but not white rot. Here, our discovery suggested that the ligninolytic oxidases may outcompete electrons and act as a “brake disc” of Fenton cycles; thus, the non-enzymatic oxidation was to some extent suppressed in white rot fungi. On the other hand, during brown rot evolution the loss of these “brake” genes allowed fungi to accelerate Fenton cycles for oxidative attacks. To validate this, we have built a genetic manipulation system in brown rot fungus *G. trabeum* (77), and recently we have tested the *in vivo* functions of laccase and ligninolytic peroxidases and their influences on Fenton cycles.

### Conclusion and future directions

Brown rot fungi adopted a Fenton mechanism to oxidatively decompose lignocellulose to kick-start the wood decay process, but the biological pathways involved, and their driving enzymes remain unclear. In this work, we assessed an enzyme-synthesizing method based on a plant-based cell-free expression system, validated its adequacy for characterizing fungal enzymes, and demonstrated that the cell-free expression method could be a promising alternative for fulfilling the increasing need for functional studies of fungal enzymes. Specifically, this method enabled us to successfully characterize the biochemical functions of two intracellular oxidoreductase enzymes [benzoquinone reductase (BQR) and ferric reductase (FRD)] and two extracellular degradative enzymes [α-L-arabinofuranosidase (ABF) and heme-thiolate peroxidase (HTP)] that were co-expressed at the oxidative step in *R. placenta*. Uncovering their catalytic features in the context of relevant brown rot environments allowed us to craft a degradative pathway model, deciphering the roles these enzymes play during early brown rot (Fig. 5).

Notably, in the model we described some new features of brown rot enzymes that allow their interactions with Fenton cycles. For example, we described an ABF possessing the extraordinary ROS resistance characteristics, which demonstrated that new intrinsic features may have been co-evolved in brown rot GH enzymes to cope with the Fenton mechanism. Also, we described the possible roles of ligninolytic oxidases (HTP and laccase) played in redirecting electron flows of Fenton cycles and suppressing the production of ROS radicals, which provided an alternative view to interpreting the roles of these enzymes played during the evolution of brown rot fungi. Even though additional brown rot enzymes with the early decay stage expression evidence would still need to be further characterized to evaluate their synchronous involvement in the Fenton mechanism. With this regard, the optimization of the cell-free expression system might be required to further improve the protein expression throughput. At the same time, gene-editing tools will be required to interrogate the *in vivo* functions of the key enzyme players in the degradation model. Taken together, experimental validation of enzyme functions *in vivo* and *in vitro* will be an important asset for better modeling and characterization of the biogeochemical cycles in which brown rot fungi are involved for future biotechnological applications.

## MATERIALS AND METHODS

### Target gene identification by RNA-seq

Target genes of this study were selected based on our previous RNA-seq studies [Gene Expression Omnibus database GSE84529 (6, 16)], in which the transcriptomic data at different decay stages were measured as the brown rot fungi, *R. placenta* (previously *Postia placenta*) and *G. trabeum*, decay a wood wafer. In the experimental setup, an aspen wood wafer was progressively colonized and decayed by fungal mycelia, allowing us to segregate, at a fine spatiotemporal scale, early from advanced decay stages. In this way, the decay stage-dependent genes were pinpointed by DEG (differentially expressed gene) analysis, and those unique to brown rot phenotype were further screened by cross-species comparisons of orthologous genes (6). Among these, four conserved, early-stage brown rot CAZymes were identified and selected in this study for characterization. These include two redox enzymes (a benzoquinone reductase and a ferric reductase) and one phenol oxidase (a heme-thiolate peroxidase) that are significantly (FDR < 0.05) upregulated at the early decay stage, and one synchronistic side-chain acting α-L-arabinofuranosidase that has high expression levels (FPKM > 100) and is important to early wood deconstruction. Corresponding genes’ IDs in *R. placenta* were reported as annotated by JGI database MAD 698R v1.0 (https://mycocosm.jgi.doe.gov/Pospl1/Pospl1.home.html) in the results. FPKM levels (Fragments Per Kilobase of transcript per Million mapped reads) of these genes were presented in Fig. 1, along with the FDR (false discovery rate of *P* values) values for significant difference analysis. Their enzymatic functions were investigated by *in silico* search of functional domains, followed by cell-free protein expression and biochemical assays, as explained below.

### Protein function prediction

The coding sequences (CDS) of candidate genes were first re-annotated with BRAKER1 (78) by integrating the RNA-seq data, and the newly annotated CDS was compared against the old gene model at JGI database using Integrative Genomics Viewer IGV 2.3 (79) to validate the alignment of RNA-seq reads onto the exons. New gene models were then accepted for protein function annotation, gene synthesis, and enzyme expression and characterization (Fig. S1). After translated into amino acid sequences, the protein homolog search and function prediction against structures in PDB databank were conducted with HHpred (https://toolkit.tuebingen.mpg.de/tools/hhpred). The predicted functions were listed in Table 1, which was then used to guide the candidate selection for cell-free protein synthesis, as well as the following enzyme assays.

### Cell-free protein synthesis

To conduct the cell-free protein synthesis, genes were custom synthesized (GenScript Biotech, NJ, USA) into compatible cell-free expression vector pEU-E01-MCS (80). The cell-free reactions were then carried out using Wheat Germ Protein Research Kit WEPRO7240 (CellFree Sciences Co., Ltd., Yokohama, Japan) according to the manufacturer’s guidelines and established protocols (36). Briefly, the reactions were first screened out at a 55 µL scale, by supplementing with pre-charged FluoroTect Green lysine tRNA (Promega, WI, USA) to test for protein expression and solubility. In short, for soluble proteins, 2 µL of mRNA was combined with 0.7 µL of FluoroTect Green_Lys_ (Promega) and 2.5 µL of wheat germ lysate and transferred under 50 µL of SUBAMIX buffer for continuous translation overnight. For two proteins (g9701 and g11221), the translation reactions were also supplemented with 2 µL of 27 mg/mL nanodiscs. Nanodiscs were made in-house from membrane scaffold protein 1E3D1 (Sigma, M7074) and DMPC lipids (Sigma, P7930) as described previously (81). To check for protein solubility, the crude mixture of each translation reaction was further split in half. One part (referred to as “crude”) was kept as it is and another part was centrifuged at 20,000 × *g* for 15 min. The pellet was discarded, and the supernatant was saved as a “soluble” fraction. Both “crude” and “soluble” fractions were then loaded on 10% SDS-PAGE gels and imaged using Typhoon FLA 9500 scanner from GE Healthcare (exc/em set for Alexa488). All cell-free reactions were then scaled up to 220 µL volumes and repeated without the addition of FluoroTect Green to produce native (unaltered) proteins. The obtained crude mixtures were used for subsequent functional characterization and enzymatic reactions. A cell-free reaction conducted at the same conditions but in the absence of pEU vector was used as a negative control. All crude protein mixtures were snap-frozen in liquid nitrogen and stored at −80°C.

### Enzyme activity evaluation

Cell-free expressed proteins were delivered from the Pacific Northwest National Laboratory to the University of Minnesota on dry ice and were stored at −80°C upon receipt and before enzyme assays. All enzyme assays and characterization tests were evaluated as three replicates, and the results were represented as the mean values ± standard deviation (SD).

Oxidoreductase activities were measured, as below, for the cell-free expressed protein products based on the predicted enzymatic functions of the candidate genes. Benzoquinone reductase (BQR) activity was evaluated by measuring the consumption of nicotinamide adenine dinucleotide phosphate (NADPH), while it catalyzes the reduction of 2,6-dimethoxy-1,4-benzoquinone (2,6-DMBQ), as described previously (82). Ferric reductase (FRD) activity was measured by monitoring the production of Fe^2+^, generated through reducing Fe^3+^, using Ferrozine as reported previously (83). Heme-thiolate peroxidase (HTP) activity was evaluated by oxidizing ABTS with H_2_O_2_ as the oxidant (84). The specific reaction conditions used for measuring enzyme activities, including the reaction mixture, pH, temperature, and others, were listed in the supplementary materials. The α-L-arabinofuranosidase (ABF) activities were measured by cleaving the 4-nitrophenyl-α-L-arabinofuranoside substrates, followed by measuring the release of 4-nitrophenol (85). Enzyme activities (U) of the cell-free expressed proteins were reported as the amount of product produced, or substrate consumed (µM) per time (min) at the conditions specified for each enzyme.

### Enzyme modeling

Protein sequences were retrieved from the Joint Genome Institute (JGI) database (https://mycocosm.jgi.doe.gov/mycocosm/home) and were re-annotated with BRAKER1 as in our previous work (6), and their 3D structures were modeled using the software MODELLER in its toolkit version available on the Max Planck Institute for Developmental Biology website (https://toolkit.tuebingen.mpg.de/tools/modeller) (ABF, HTP, and BQR) or AlphaFold2 (https://colab.research.google.com/github/sokrypton/ColabFold/blob/main/beta/AlphaFold2_advanced.ipynb#scrollTo=xkCcitL6qdNS) (FRD) with the default parameters. Protein structures were then validated using Procheck, Prosa, Verify 3D, and ERRAT as described previously (86). Enzyme transmembrane topologies were determined with the tool DeepTMHMM (https://dtu.biolib.com/DeepTMHMM).

### Catalytic characteristics of enzymes

The effects of temperature, pH, inhibitors, and ROS on the performance of cell-free expressed enzymes were characterized. The optimum catalytic conditions of the enzymes were evaluated for the pH range of 3.0–8.0 and the temperature range of 20–70°C. Enzyme tolerance to pH or temperature was determined by incubating enzyme samples in different pH or temperature environments for 3 h for analyzing the residual activities. The effects of different metal inhibitors and ethylenediaminetetraacetic acid (EDTA) were evaluated at a concentration of 10 mM, while NaN_3_ was tested at a concentration of 1 mM (87). Enzyme tolerance to ROS was measured by incubating enzyme samples with the mixture of 1 mM Fe^2+^ and 5 mM H_2_O_2_ that can trigger the production of hydroxyl radicals through Fenton reaction (47). After 1 h treatment at room temperature, the excess H_2_O_2_ was degraded by adding catalase and incubating for another hour at 30°C, followed by determining the residual activities, as above. Commercial laccase (Sigma, SIAL-SAE0050-200 mU) was also used for comparison purposes.

K_m_ and V_max_ values for enzymes HTP, ABF, BQR, and FRD were determined by measuring the catalytic kinetics on the substrates of 50–900µM ABTS, 0.1 mM-2.3 mM 4-nitrophenyl-α-L-arabinofuranoside, 30–570 µM 2,6-DMBQ, and 40–400 µM Fe^3+^, respectively.

### Quinone redox cycling

The roles of brown rot enzymes BQR, HTP, and laccase and their interactions in driving the quinone redox cycling for ROS production were investigated. A BQR reaction mix prepared as described before (supplementary materials) containing 2,6-DMBQ (0.2 mM) was tested for its ability to reduce Fe^3+^. Briefly, 200 µL of BQR reaction mix was added to 20 µL of 50 mM acetate buffer pH 5 (w/ or w/o laccase or HTP) with 100 µL of 0.4 mM Fe^3+^ prepared in 50 mM acetate buffer pH 5.0, 50 µL of 10 mM oxalate, and 54 µL of 2 mM ferrozine. The sample was incubated for 20 min and the absorbance was measured at 562 nm. The blank for BQR consisted of 220 µL of 50 mM phosphate buffer pH 6.0 (the reaction buffer for BQR). H_2_O_2_ produced by the system was measured by the Amplex Red10 (AR10, 10-acetyl-3,7-dihydroxyphenoxazine; Thermo Fisher Scientific, USA) method, which was evaluated as highly sensitive and selective for H_2_O_2_ quantification for studying fungal wood decomposition in a previous report (88).

### Statistical analysis

Two-tailed paired *t* tests were used to establish significant differences when required, given unequal variances among the various treatments. One-way ANOVA and multiple comparisons were used for analyzing the effects of different treatments on the performance enzymes.

## SUPPLEMENTAL MATERIAL

Supplemental material aem.00122-24-s0001.docx Supplemental methods and results.

ASM does not own the copyrights to Supplemental Material that may be linked to, or accessed through, an article. The authors have granted ASM a non-exclusive, world-wide license to publish the Supplemental Material files. Please contact the corresponding author directly for reuse.
