# Supplementary material for: Unlocking the distinctive enzymatic functions of the early plant biomass deconstructive genes in a brown rot fungus by cell-free protein expression
Source: Appl Environ Microbiol. 2024 Apr 3;90(5):e00122-24. doi: 10.1128/aem.00122-24 (PMC11205865; doi:10.1128/aem.00122-24)
Supplement: Supplemental material — Supplemental methods and results. [file aem.00122-24-s0001.docx]

**Unlocking the Distinctive Functions of the Early Plant Biomass Deconstructive Stage Enzymes in a Brown Rot Fungus by Cell-free Protein Expression**

Jesus D. Castaño^1^, Irina V. El Khoury^2^, Joshua Goering^1^, James E. Evans^2,3^, and Jiwei Zhang^1*^

^1^ Bioproducts and Biosystems Engineering, University of Minnesota, Saint Paul, MN, USA, 55108

^2^ Environmental Molecular Sciences Laboratory, Pacific Northwest National Laboratory, Richland, WA, USA, 99354

^3^ School of Biological Sciences, Washington State University, Pullman, WA, 99164, USA

*Corresponding Author: Jiwei Zhang ([zhan3437@umn.edu](mailto:zhan3437@umn.edu)); +1 612-624-1761

**Supplementary material**

**I. Materials and methods for enzyme assays**

All activities were reported as the amount of product produced, or substrate consumed (µM) per time (min) at the conditions specified for each enzyme.

**(a) Benzoquinone reductase (BQR)** activity was evaluated by measuring the consumption of NADPH (Nicotinamide adenine dinucleotide phosphate) while it catalyzes the reduction of 2,6-dimethoxy-1,4-benzoquinone (2,6-DMBQ), as described previously ^1^. Briefly, 625 µL of 50 mM phosphate buffer at pH 6.0 were mixed with 200 µL of 1 mM 2,6-DMBQ prepared in DMSO, 5 µL of 1 mM flavin mononucleotide (FMN) cofactor, 20 µL of enzyme sample, and 100 µL of 5 mM NADPH. The disappearance of NADPH was followed by monitoring the absorbance at 340 nm.

**(b) Ferric reductase (FR)** activity was measured by following the reduced Fe^2+^ using Ferrozine, as reported previously ^2^. In specific, 50 µL of enzyme extract were mixed with 40 µL of 2 mM Ferrozine, 100 µL of 0.4 mM FeCl_3_, 10 µL of 3 mM NADPH, and 50 µL of phosphate buffer at pH 6.0. The reaction was incubated at 40°C and the production of Fe^2+^ was measured by monitoring the absorbance at 562 nm.

**(c)** **Heme thiolate peroxidase (HTP)** activity was evaluated by using ABTS as described before ^3^. Briefly, 15 µl of enzyme extract were mixed with 20 µl of 10 mM ABTS, 160 µl of 50 mM sodium acetate buffer pH 5, and 15 µl of 40 mM H_2_O_2_. After starting the reaction by the addition of H_2_O_2_, the oxidation of ABTS was followed at 415 nm.

**(d) p-Nitrophenyl derivative-based assays.** α-L-Arabinofuranosidase activities were measured using 4-nitrophenyl-α-L-arabinofuranoside, as previously reported^4^. Briefly, 50 µL of 100 mM acetate buffer pH 5.0, 50 µL of p-nitrophenyl-derivative, and 50 µL enzyme sample were mixed. The samples were incubated at 40°C for 1 h, and then put on ice for 5 min. The reactions were ended by the addition of 150 µL of 10% Na_2_CO_3_, and subsequently incubated for 15 minutes at room temperature. The release of p-nitrophenol was measured by monitoring the absorbance at 420 nm.

**II. Reannotation of the gene models**

**
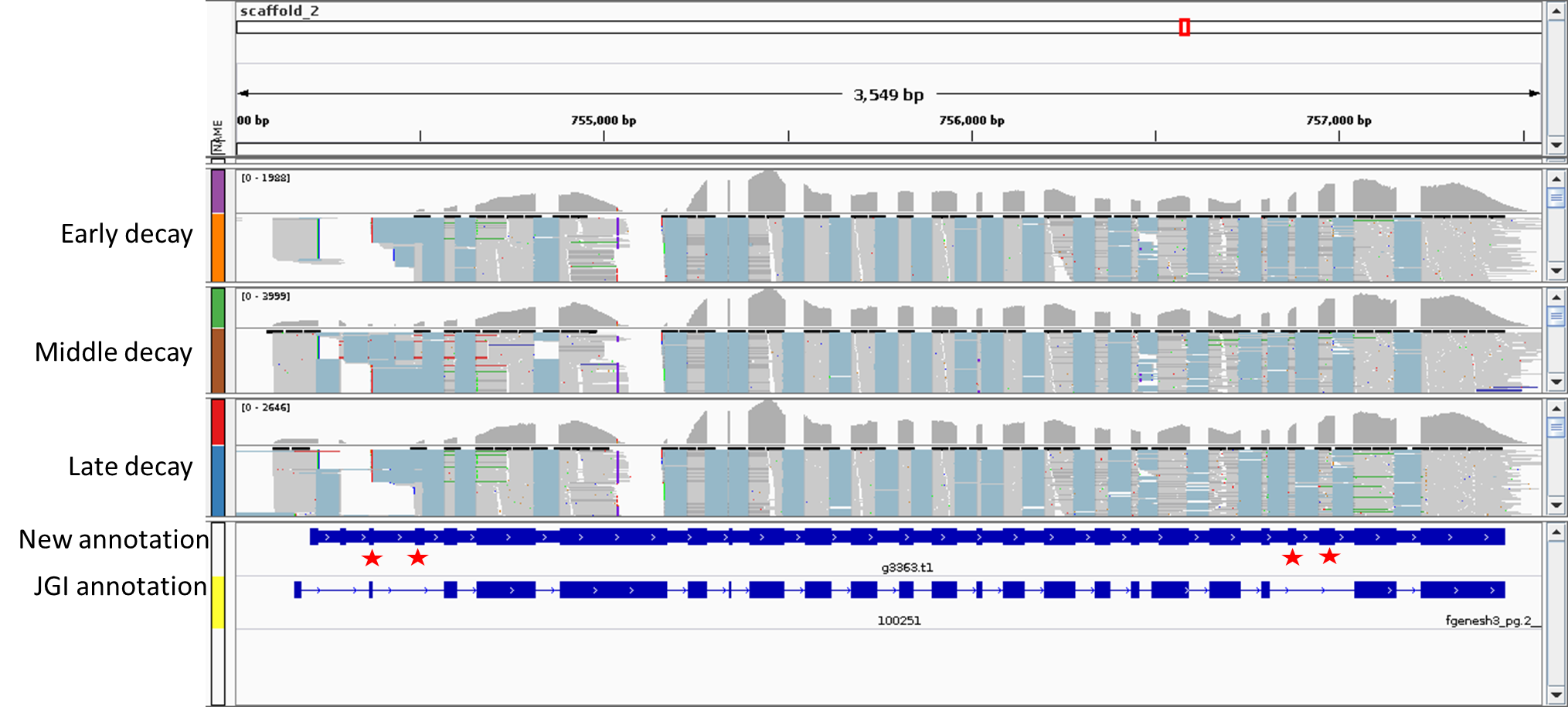
**
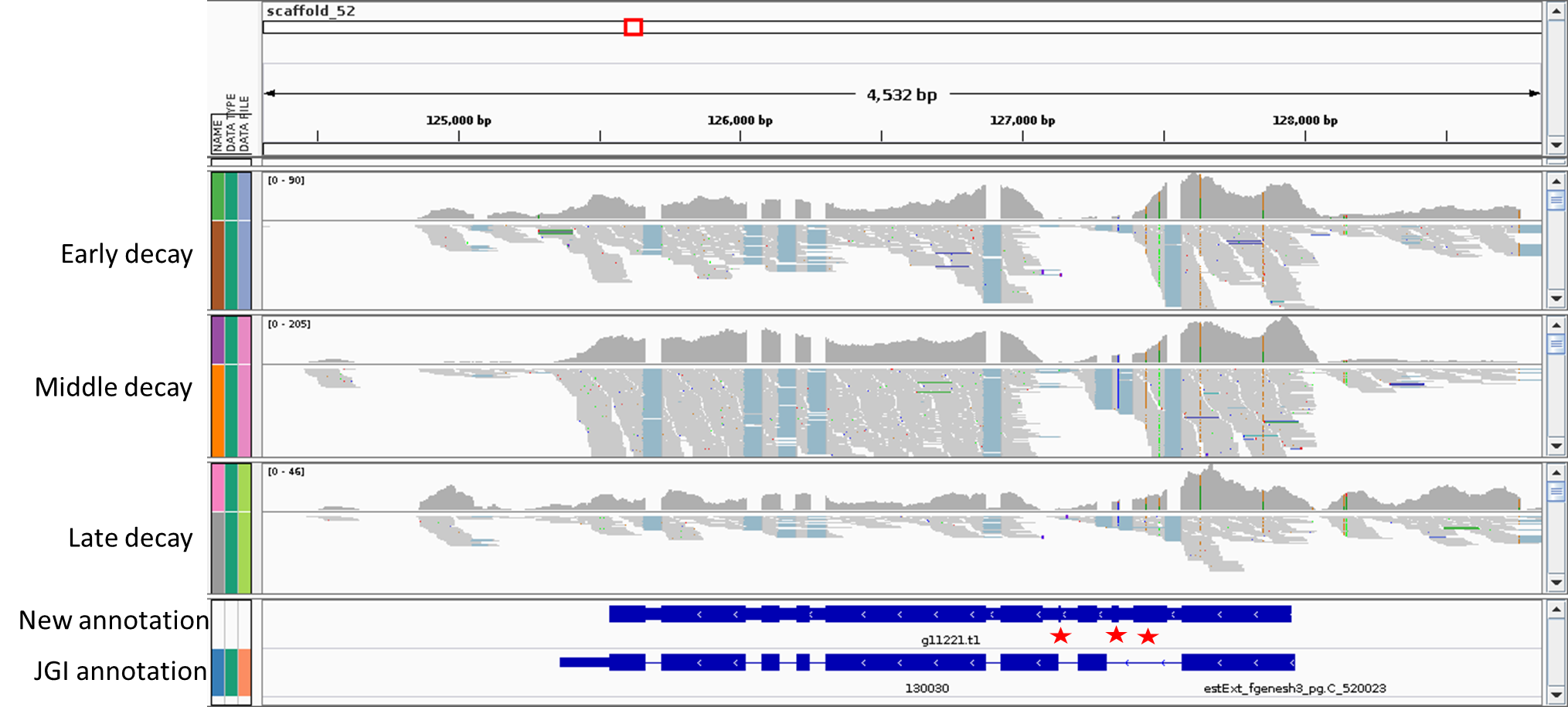


**Fig. S1.** Comparisons of JGI’s and the re-annotated gene models revealed that two genes of interest, as above, were improved by re-defining the exons and introns evidenced by RNA-seq data. Transcriptomics were measured along with three decay stages of *R. placenta*. Red stars represent the regions where the re-annotated CDS locates. Both the new IDs (up) and JGI protein IDs (bottom) were available in Table 1.

**III. Enzyme characterization results**

**
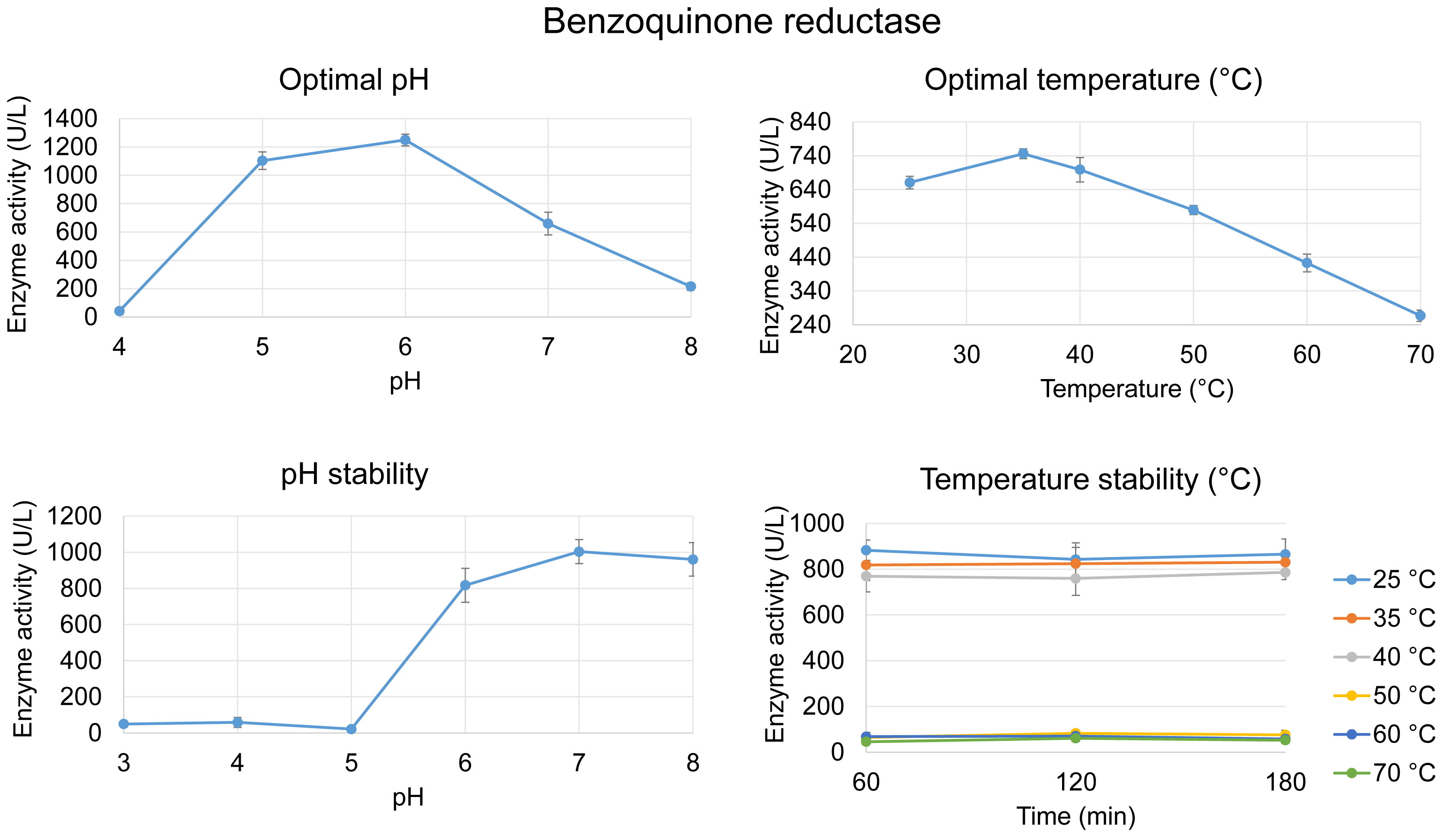
**

**Fig. S2.** Characterization results for BQR. Four parameters were studied, namely optimal pH (top left), optimal temperature (top right), pH stability (bottom left), temperature stability (bottom right).

**
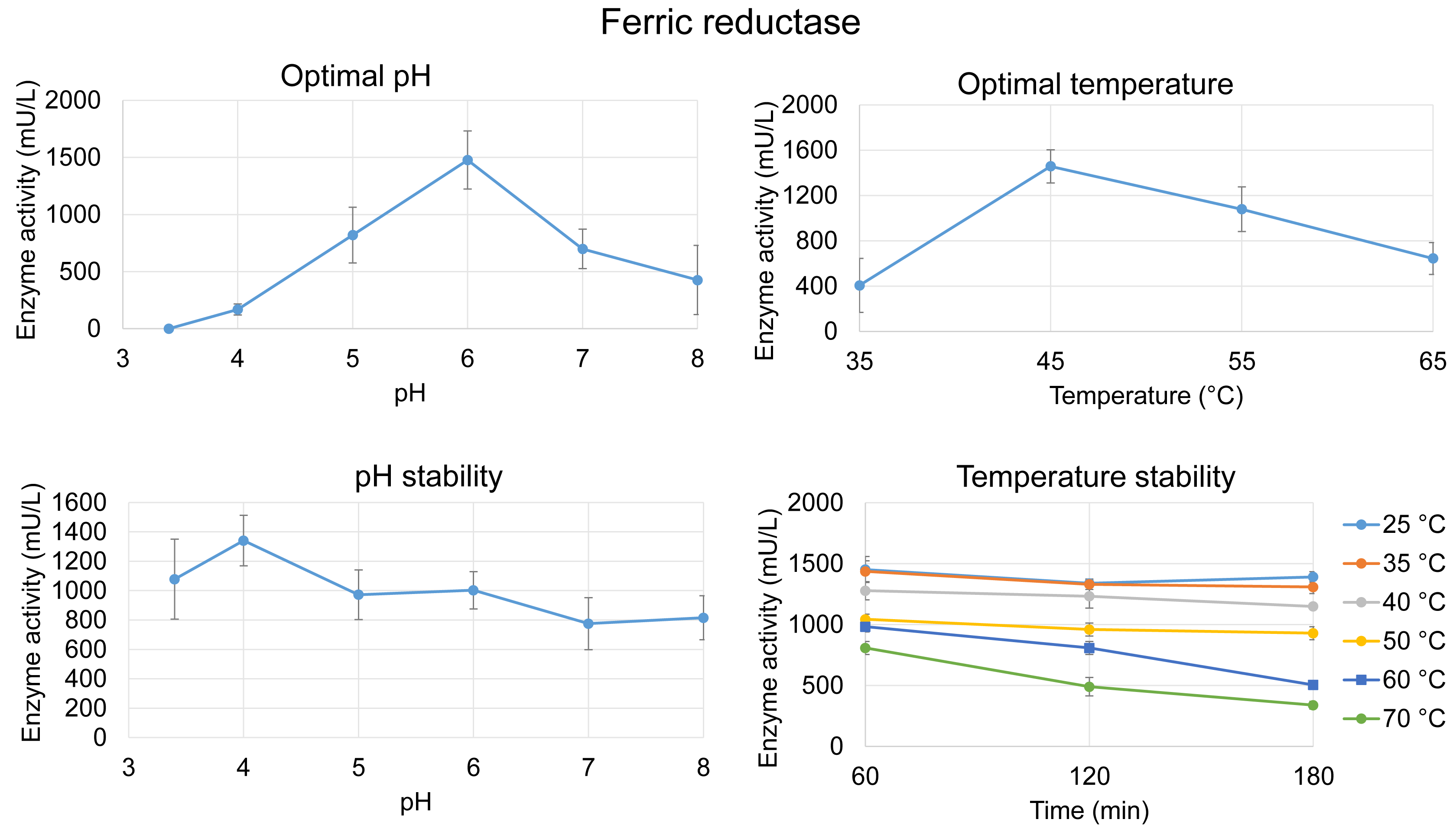
**

**Fig. S3.** Characterization results for FRD. Four parameters were studied, namely optimal pH (top left), optimal temperature (top right), pH stability (bottom left), temperature stability (bottom right).

**
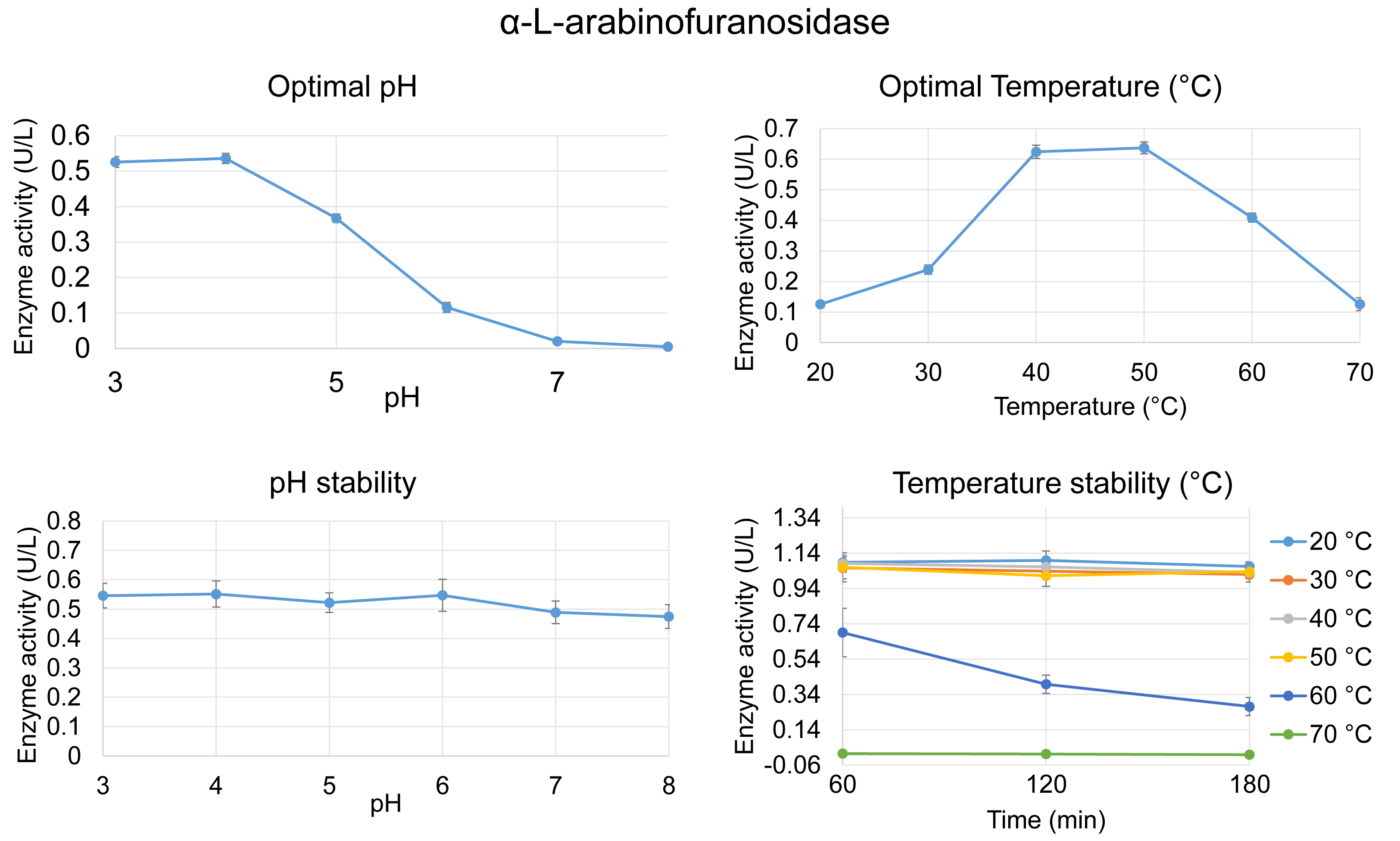
**

**Fig. S4.** Characterization results for ABF. Four parameters were studied, namely optimal pH (top left), optimal temperature (top right), pH stability (bottom left), temperature stability (bottom right).

**
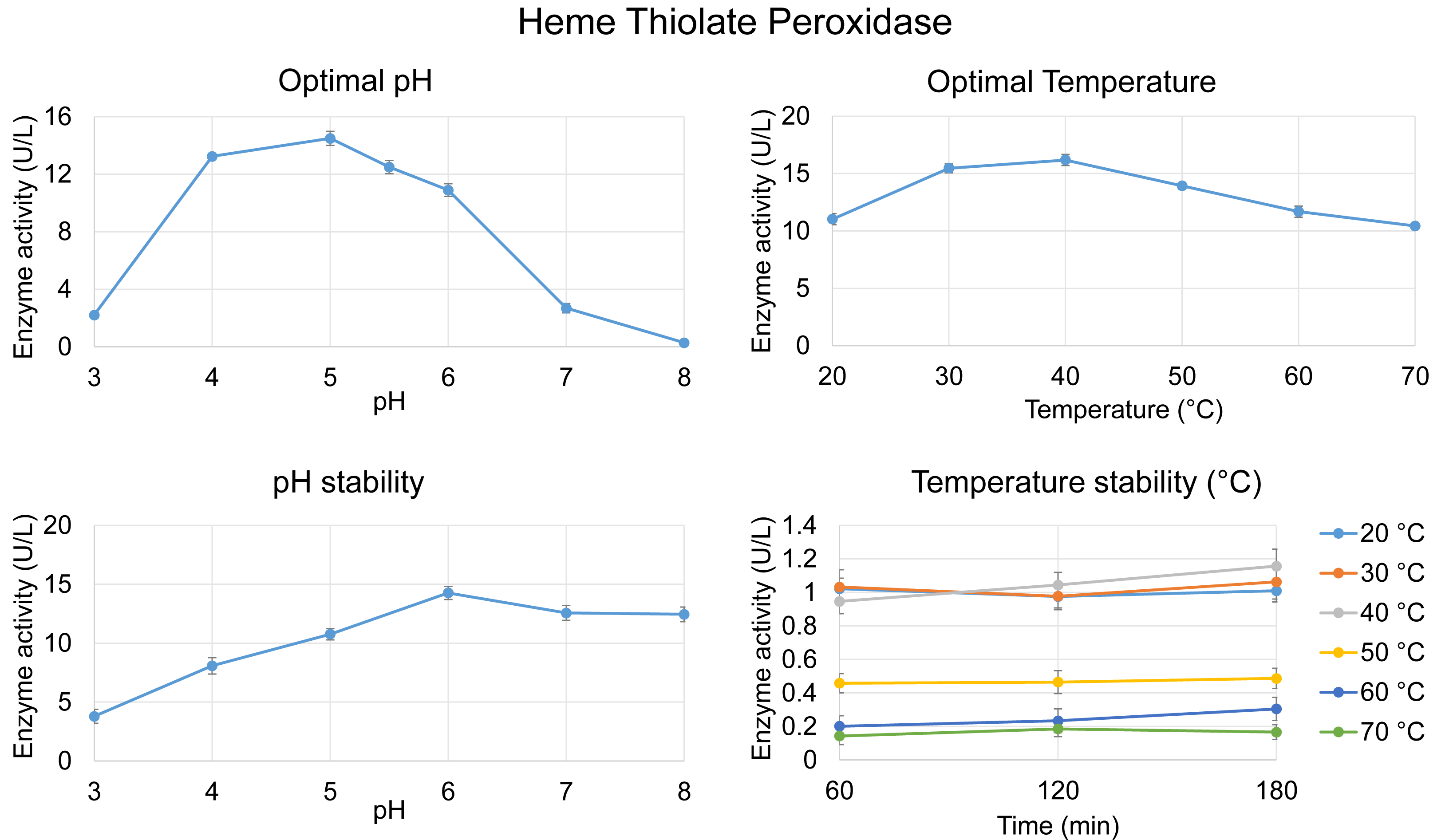
**

**Fig. S5.** Characterization results for HTP. Four parameters were studied, namely optimal pH (top left), optimal temperature (top right), pH stability (bottom left), temperature stability (bottom right).

**References**

(1) Akileswaran, L.; Brock, B. J.; Cereghino, J. L.; Gold, M. H. 1,4-Benzoquinone Reductase from Phanerochaete Chrysosporium: cDNA Cloning and Regulation of Expression. *Appl. Environ. Microbiol.* **1999**, *65* (2), 415–421. https://doi.org/10.1128/AEM.65.2.415-421.1999.

(2) Yi, Y.; Guerinot, M. L. Genetic Evidence That Induction of Root Fe(III) Chelate Reductase Activity Is Necessary for Iron Uptake under Iron Deficiency. *Plant J. Cell Mol. Biol.* **1996**, *10* (5), 835–844. https://doi.org/10.1046/j.1365-313x.1996.10050835.x.

(3) Ullrich, R.; Nüske, J.; Scheibner, K.; Spantzel, J.; Hofrichter, M. Novel Haloperoxidase from the Agaric Basidiomycete Agrocybe Aegerita Oxidizes Aryl Alcohols and Aldehydes. *Appl. Environ. Microbiol.* **2004**, *70* (8), 4575–4581. https://doi.org/10.1128/AEM.70.8.4575-4581.2004.

(4) Mallek-Fakhfakh, H.; Belghith, H. Physicochemical Properties of Thermotolerant Extracellular β-Glucosidase from Talaromyces Thermophilus and Enzymatic Synthesis of Cello-Oligosaccharides. *Carbohydr. Res.* **2016**, *419*, 41–50. https://doi.org/10.1016/j.carres.2015.10.014.
